# Development of newborn screening connect (NBS connect): a self-reported patient registry and its role in improvement of care for patients with inherited metabolic disorders

**DOI:** 10.1186/s13023-017-0684-3

**Published:** 2017-07-19

**Authors:** Yetsa Osara, Kathryn Coakley, Aishwarya Devarajan, Rani H. Singh

**Affiliations:** 1 Metabolic Genetics and Nutrition Program, Emory University, Atlanta, GA USA; 2 Department of Individual, Family and Community Education, University of New Mexico, Albuquerque, NM USA; 3 Division of Genetics and Genomics, Boston Children’s Hospital, Boston, MA USA; 4 Department of Human Genetics, Metabolic Genetics and Nutrition Program, Emory University, 2165 North Decatur Road, Decatur, GA 30033 USA

**Keywords:** Patient registry, Newborn screening, Phenylketonuria, Maple syrup urine disease, Tyrosinemia, Metabolic disorders, NBS Connect

## Abstract

**Background:**

Newborn Screening Connect (NBS Connect) is a web-based self-reported patient registry and resource for individuals and families affected by disorders included in the newborn screening panel. NBS Connect was launched in 2012 by Emory University after years of planning and grassroots work by professionals, consumers, and industry. Individuals with phenylketonuria (PKU), maple syrup urine disease (MSUD) or tyrosinemia (TYR) have been recruited through distribution of outreach materials, presentations at parent organization meetings and direct recruitment at clinic appointments. Participants complete online profiles generating data on diagnosis, treatment, symptoms, outcomes, barriers to care, and quality of life. Resources such as education materials, information on the latest research and clinical trials, recipes, interactive health tracking systems, and professional support tools are described. In addition, to examine the ability of NBS Connect to generate data that guides hypothesis-driven research, data pertaining to age at diagnosis, bone health, and skin conditions in individuals with PKU were assessed. The objective of this paper is to describe the development of NBS Connect and highlight its data, resources and research contributions.

**Results:**

In September 2016, NBS Connect had 442 registered participants: 314 (71%) individuals with PKU, 68 (15%) with MSUD, 20 (5%) with TYR, and 40 (9%) with other disorders on the NBS panel. Age at diagnosis was less than 4 weeks in 285 (89%) of 319 respondents to this question and between 1 month and 14 years in 29 (9%) individuals. Of 216 respondents with PKU, 33 (15%) had a DXA scan in the past year. Of 217 respondents with PKU, 99 (46%) reported at least one skin condition.

**Conclusions:**

NBS Connect was built and refined with feedback from all stakeholders, including individuals with inherited metabolic disorders. Based on patient-reported data, future studies can be initiated to test hypotheses such as the relationship between PKU and skin conditions. Patient registries like NBS Connect can inform hypothesis-driven research, contributing to knowledge generation and following the current trend in moving from traditional medicine towards evidence-based practice. NBS Connect will help clinicians understand long-term outcomes of rare disorders, contributing to better patient care and quality of life.

## Background

Newborn screening (NBS) is an early-detection tool to identify inherited disorders using a minimal amount of blood from a heel prick in newborn infants. Developed and initiated in the United States during the 1960s with screening for Phenylketonuria (PKU),NBS has demonstrated its public health value by identifying thousands of infants with an expanding number of disorders [1]. Early detection allows prompt medical treatment to prevent devastating long-term effects of many disorders included in the newborn screening panel [2].

As the number of individuals with disorders detected by NBS increases, the U.S. Centers for Disease Control and Prevention (CDC) and the Health Resources and Services Administration (HRSA) have recognized the need for high-quality research and clinical expertise to maximize patient care [3]. As a result, NBS initiatives were formed such as the Southeast Regional Newborn Screening & Genetics Collaborative (SERC), established through HRSA. The primary aims of SERC are to promote translation of healthcare research into practice and to improve care for individuals with heritable disorders in the southeast region of the United States [4]; however, we hope SERC activities have a national and global reach

Development of the Newborn Screening Connect (NBS Connect) patient registry is one of SERC’s efforts to enhance patient care, add to clinical research, and promote translational medicine [4]. Patient registries are an organized and secure method of collecting information on individuals with various disorders. In most registries, data are collected by study coordinators who enter information into an online or computer-based system. NBS Connect, however, is a self-reported patient registry that involves data entry directly by patients. Similar patient registries demonstrate the utility of self-reporting including DuchenneConnect for individuals with Duchenne and Becker muscular dystrophy and Remudy, a Japanese registry for muscular dystrophy [5, 6]. Data published from DuchenneConnect show the clinical presentation, medication use, and cardiac abnormalities in affected patients [5]. Remudy uses its database to recruit eligible patients as participants in clinical trials [6]. Self-reported registries represent a valuable tool for collecting data from numerous individuals with rare disorders, and NBS Connect is the first self-reported patient registry for disorders included on the NBS panel, with an initial focus on inherited metabolic disorders (IMD).

The purpose of NBS Connect is to collect and analyze primary data on diagnosis, treatment, symptoms, outcomes, and barriers to care in disorders included on NBS panel. NBS Connect also provides professional support resources for patients and families. The registry offers unique features such as educational materials, low protein recipes analyzed by registered dietitians, interactive health tracking tools for data visualization, information about the latest research and clinical trials, opportunities to connect with experts and a forum for patients and their parents to connect with each other. Patients with a wide range of metabolic disorders are able to register; however, disorder-specific profiles have been developed for patients with PKU, Maple Syrup Urine Disease (MSUD) and Tyrosinemia (TYR). NBS Connect will continue to expand to include other disorders in the recommended uniform screening panel (RUSP) endorsed by the American College of Medical Genetics and Genomics (ACMG) [7]. Many of these disorders are rare and do not have the infrastructure of support groups or independent registries to connect patients with each other, clinicians, and researchers. NBS Connect aims to fill this tremendous gap. We hope to create a common social platform for individuals and families affected by rare disorders and provide a reliable source of publically available data to enhance the knowledge base and improve treatment.

The objectives of this manuscript are to describe the development of the NBS Connect patient registry and highlight its data, resources and research contributions.

## Methods

### Development of NBS connect

**Fig. 1:**
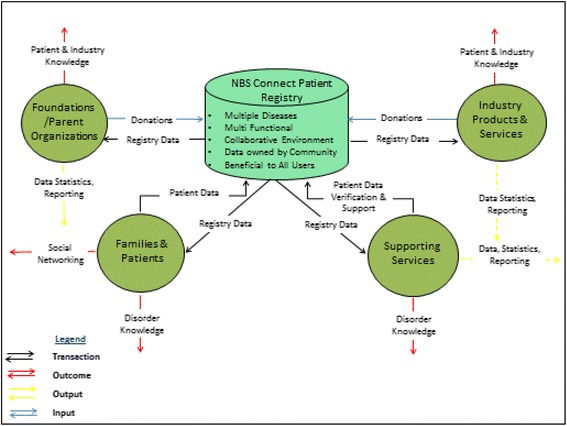
NBS Connect Developmental Framework

The NBS Connect project was approved by Emory University’s Institution Review Board in 2011. The registry was developed over years of planning and grassroots efforts from a collaboration of professionals, patients and parents. The original idea and initial planning of the registry was led by professionals with solicited input from patients, and parents. The NBS Connect operational team included the principle investigator (PI), project manager, dietitians, and web development team (PatientCrossroads), who were responsible for developing the NBS Connect framework into a functional platform. NBS Connect is the product of combined efforts of all team members, as well as auxiliary stakeholders such as parent support groups, industry sponsors, and funding agencies. The developmental framework for NBS Connect is illustrated in [Fig. 1].

#### Patient focus groups and summit meetings

After the idea and framework for a self-reported patient registry for IMD were developed by the project PI, SERC hosted several informal focus groups and two formal summit meetings to facilitate collaborations between clinical experts and parents of individuals with metabolic disorders. These meetings were held to engage patient support organizations, assess the needs of the patient community and ensure a consumer-friendly registry.

The first summit, held in February 2010, included parents of children with metabolic disorders and members of patient support organizations. Using a questionnaire tool, participants provided feedback on the registry’s proposed purpose, features, mission, and sustainability. Information concerning the accessibility, value, necessity and usability were also collected to build a user-friendly interface.

A second SERC summit meeting was held in May 2010 and was attended by representatives from the Fatty Acid Oxidation Disorder (FAOD) support group, MSUD family support group, National PKU Alliance (NPKUA), Organic Acidemia Association (OAA), Parents of Galactosemic Children (now called the Galactosemia Foundation); as well as representatives from Genetic Metabolic Dietitians International (GMDI), National Society of Genetic Counselors (NSGC), Emory University and DuchenneConnect. Summit meeting participants attended a presentation about NBS Connect and contributed to discussions on the purpose of the patient registry, its target population, outreach activities and sources of funding. In addition, participants rated their overall perception of the NBS Connect registration process, access, user-friendliness, navigation and privacy protection on a scale of 1 (poor) to 5 (great).

The registry currently receives federal funding from HRSA, supplemented by funds through industry grants. In addition, the project has been presented to Newborn Screening Translational Research Network (NBSTRN) funded to ACMG through the National Institutes of Health (NIH) as an activity for NBS long-term follow-up and to complement the existing clinician-based registry for long-term sustainability.

#### Profile development and data collection

##### Disorder-specific profiles

Once the NBS Connect technology infrastructure was developed by PatientCrossroads, profiles were developed to collect patients’ self-reported data. The online patient profile consists of two sections: a basic profile which includes demographic questions such as family history, development and social history, insurance, and research interests; and a disorder-specific profile which includes questions about diagnosis, genetic testing, treatment, and diet management. Both profiles were designed by the HRSA/SERC team of dietitians, medical professionals and genetic counselors who provided content matter expertise. Once basic and disorder-specific profile questions were finalized, they were published online and a beta test analysis was conducted.

##### Beta test analysis

For each disorder-specific profile, a beta test was conducted to determine the ease of completing questions, readability, and uniformity. As part of the beta test, volunteers from parent organizations were asked to register on NBS Connect and complete the basic profile and the relevant disorder-specific profile. After completion, volunteers were requested to provide feedback through an online survey. The first beta test was used to collect comprehensive feedback on the NBS Connect website and the PKU profile; subsequent beta tests collected feedback on the MSUD and TYR profiles.

##### Data collection

After beta testing, individuals with PKU, MSUD or TYR were recruited to join the NBS Connect registry through distribution of outreach materials to metabolic clinics, presentations at parent organization meetings and direct recruitment at clinic appointments. Participation in the registry is voluntary and all participants provide electronic informed consent and review the privacy notice and terms and conditions. Once consent is obtained, participants select their disorder and complete the basic and disorder-specific profiles. Individuals who are at least 18 years old can register themselves, while persons under 18, or under the care of a legal guardian, are registered by a parent or guardian. Participants that turn 18 are contacted via email and encouraged to take ownership of their profile. Doing so allows them to update log in credentials, review their information and provide annual updates from their perspective. Participants also have the option of letting their caregiver maintain the profile on their behalf. Families with more than one affected individual can choose to link more than one profile and use the same log-in information to access multiple family profiles. All registered participants are emailed a reminder to update information annually.

Profile questions regarding demographics and diagnosis are mandatory; however, other questions are optional. Various answer choices are provided for each question and an “other” response is available. NBS Connect is committed to protecting the identity of participants. All patient-entered data are de-identified and stored with a code instead of the patient’s name, removing all protected health information (PHI). Through the registry’s online Health Insurance Portability and Accountability Act (HIPAA) authorization form, participants are asked to indicate their sharing preferences and are given the option to opt out of information sharing. They are also permitted to revoke their permission at any time during participation in the registry.

Access controls through data encryption ensure that there is no accidental or unauthorized disclosure of data. All PatientCrossroads applications dealing with patient or HIPAA protected data are operated over Secure Sockets Layer (SSL) Uniform Resource Locator (URL) encoding. This prevents access by other internet users that may be ‘spoofing’ or otherwise attempting to intercept or reverse engineer the registry data. Integrity measures are also taken to ensure data are not unintentionally or maliciously altered. Only users with appropriate credentials can access identifiable and encrypted data. Access to the NBS Connect database or servers is limited to Virtual Private Network (VPN), requiring 4 levels of passwords; all VPN transmissions are encrypted with SSL. Accountability measures track actions or behaviors of users by auditing how data are accessed.

### Example of hypotheses generation

The NBS Connect operational team can query the database to retrieve specific information, based on a preferred search criterion. Queries generate the specified data set in Microsoft excel format for download and analysis. Other patient data can be aggregated and explored using charts and graphs through the NBS Connect interface. To examine the ability of data collected through NBS Connect to identify new research questions, age at diagnosis, bone health and skin conditions in individuals with PKU were assessed in 2016 [Table 1]. The queries on bone and skin health were selected based on reports of issues which had no consensus on evaluating and monitoring in PKU [8–10].

**Table 1:** Questions and response options for Age at Diagnosis, Bone Health and Skin Disorders

Question and Answers for the data sets			
How old was the affected person at diagnosis?			
Unknown	10 - 12 months	9 years	18 years
Prebirth	1 year	10 years	19 years
	2 years	11 years	20 years
less than 1 week	3 years	12 years	21 years
1 - 2 weeks	4 years	13 years	22 years
3 - 4 weeks	5 years	14 years	23 years
1 - 3 months	6 years	15 years	24 years
4–6 months	7 years	16 years	25 years
	8 years	17 years	> 25 years
7 - 9 months			
Has the affected person had bone density testing (DXA) done within the last year?			
Yes - Normal result			
Yes - Abnormal result			
Unknown			
No			
Has the affected person ever been diagnosed with a bone disorder? (check all that apply)			
No			
Unknown			
Bone fractures			
Curvature of the spine			
Osteomalacia			
Osteopenia			
Osteoporosis			
Rickets			
Scoliosis			
Other			
Has the affected person had symptoms of a skin condition? (check all that apply)			
No			
Unknown			
Herpes Simplex Virus			
Herpes Zoster			
Hives			
Psoriasis			
Eczema			
Rosacea			
Acne			
Warts			
Seborhheic Dermatitis			
Other			

Bone density is hypothesized to be low in patients with PKU, but cause is unknown [11]. To examine clinical monitoring and status of bone health in PKU, the registry collects data on frequency of dual energy x-ray absorptiometry (DXA) scans and results (normal or abnormal) as reported by participants. The database was queried to report the number of individuals with PKU who had a DXA scan within the last year, result, and prevalence of those diagnosed with a bone disorder.

The registry also collects data on skin conditions, an underreported indicator of health among individuals with PKU. The database was queried to retrieve the number of individuals who report skin conditions and the types of skin conditions reported. Individuals with skin conditions were further classified by the number of skin conditions present, with some reporting more than one condition.

### Resources and professional support

Collection and analysis of data through NBS Connect are major advantages to researchers; however, part of developing and creating a unique registry included the provision of several resources and professional support tools for patients. Resources include *Kitchen Connection*, a collection of low protein recipes curated and analyzed by registered dietitians. This resource consists of multiple dish categories with nutrition information calculated per serving including calories and amino acids relevant to NBS Connect disorders (phenylalanine, leucine and tyrosine).

The *Ask an Expert* feature is a professional support tool through which participants can submit questions to professionals and clinicians regarding the NBS Connect registration process, genetic testing, diet management, recipes, clinical trials, resources and other general comments. Questions are answered within two business days by subject matter experts including dietitians, the project manager or project director.

*Locate a Clinic* is a resource provided to assist parents and caregivers in finding dietitians and metabolic clinics by state. This tool gives families a list of metabolic clinics and associated contact information for clinics close to their location.

All registered NBS Connect members have access to information about current domestic and international research and clinical trials. The news section provides an excellent source of up-to-date information and events from the metabolic community.

The *Discussion Forum* is a feature of NBS Connect that provides a safe and secure area where registered participants can share and learn from each other’s experiences. There are sections for topics such as *Recent Diagnosis* where parents of participants can share their experience of learning their child’s diagnosis, *Medical Issues* where participants can discuss any health challenges they or their child are experiencing, *Behavioral Issues* to share questions and concerns about behavioral issues, and an *Education and Therapy* section to discuss learning and educational issues as well as interventions and therapy. A *Social Connections* section was also added to encourage users to connect and stay in touch with one another. All forum discussions are moderated by the project coordinator. Empowering individuals, connecting with practitioners, and promoting health and wellness in individuals with IMD are all priorities of the registry. The results presented here include qualitative analyses to explain the development, implementation and research potential of NBS Connect.

## Results

### Development of NBS connect

NBS Connect was launched in 2012 with NBS-PKU Connect as the pilot condition. The feedback received from discussions at focus groups and summit meetings was utilized in the development of the disorder-specific profiles, web display, and overall utility of NBS Connect. With the success of PKU, the next phase was launched in 2013 to include NBS-MSUD Connect. In 2015 the NBS-TYR Connect phase was launched as well.

The overall reaction to NBS Connect from focus group participants was rated “great” based on the comprehensive nature of the registry, continuous monitoring and data curation by the operations team. The majority of participants rated the registry as highly beneficial to patients with metabolic disorders. On average, the navigation, registration, accessibility and user-friendliness of NBS Connect were rated “good”, specifically the order of questions, inclusion of additional response options to cover unique circumstances, and arrangement of content on the homepage. Data security and participant confidentiality was rated as secure, a “4” on a scale of 1 to 5, with 5 being “very secure”. Challenges reported while using the registry were “crowded display”, lack of “warm feeling”, and the limited ability to reach populations who do not have internet access. Changes suggested by participants were to state the data security level of the website and to list types of individuals who may access de-identified data. These reported challenges and changes were addressed before the launch of NBS Connect in 2012. Overall, the parent group and summit participants recognized NBS Connect as a comprehensive and centralized registry for patients with metabolic disorders.

### Questionnaire development and data collection

#### Disorder-specific profile

The disorder-specific profiles for PKU, MSUD and TYR were developed and launched for testing in December 2011, August 2013 and July 2015 respectively. The majority of parents who participated in testing strongly agreed that both the general NBS Connect profile and each disorder-specific profile for PKU, MSUD and TYR were clear, concise, and easy to use. Parents also strongly agreed that they would recommend NBS Connect to other affected individuals and families. The disorder-specific profiles were modified based on feedback received to include questions about diet compliance, reasons for non-compliance, and inclusion of additional answer choices for age ranges including newborns younger than one week.

#### Data collection

As of September 2016, the NBS Connect patient registry had 442 registered participants: 314 (71%) individuals with PKU, 68 (15%) individuals with MSUD, 20 (5%) individuals with TYR and 40 (9%) individuals with other disorders included on the NBS panel for which profiles are not yet developed. Enrolled participants represent forty five states and twenty one countries. [Table 2] summarizes the characteristics of the participants in the patient registry.

**Table 2:** Characteristics of Registered Participants on NBS Connect

Characteristics	N (%)	Disorder-specific %
All Registered Participants	442 (100)	
Phenylketonuria	314 (71)	
< 18 years	182	58
≥ 18 years	132	42
Males	127	40
Females	187	60
Maple Syrup Urine Disease	68 (15)	
≤ 18 years	38	56
> 18 years	30	44
Males	37	54
Females	31	46
Tyrosinemia	20 (5)	
≤ 18 years	16	80
> 18 years	4	20
Males	12	60
Females	8	40
Other Disorders	40 (9)	-

### Example of hypotheses generation

**Fig. 2:**
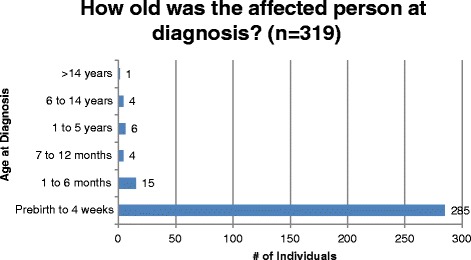
Distribution of individuals based on the age at diagnosis

**Fig. 3:**
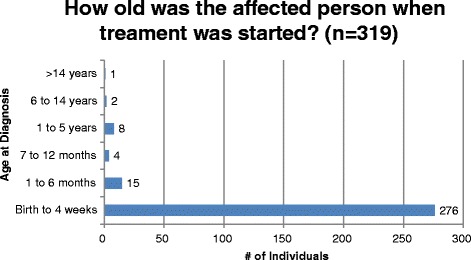
Distribution of individuals based on the age at which treatment was started

**Fig. 4:**
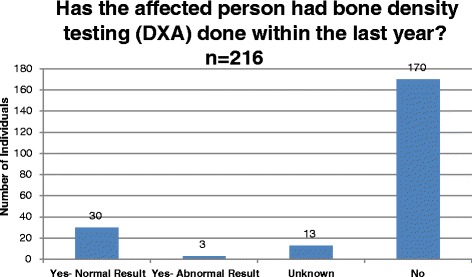
Distribution of PKU participants who reported on DXA testing

**Fig. 5:**
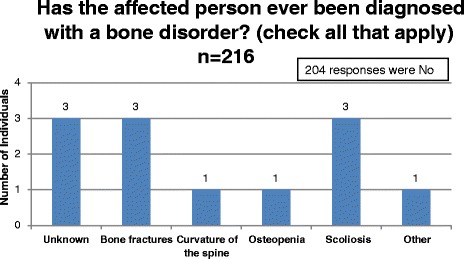
Distribution of PKU participants who reported on Bone Diagnosis

**Fig. 6:**
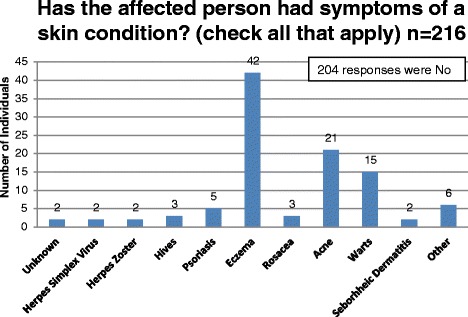
Distribution of PKU participants who reported on Skin Disorders

Among all registry participants who responded to the diagnosis question (*n* = 319), the age of diagnosis was reported to be less than four weeks in 285 (89%) individuals and between one month and 14 years in 29 (9%) individuals. The age at which treatment was started follows the same distribution as diagnosis, demonstrating the purpose of NBS, which is early treatment following a positive NBS result [2]. [Figure 2, Fig. 3]. Among 216 participants with PKU who responded to the DXA question, 33 (15%) individuals reported having a DXA scan in the past year; 30 (91%) of these were normal [Fig. 4]. The ages of participants who reported a bone scan ranged from four to 47 years. Bone fractures and scoliosis were the two most frequently reported bone disorder diagnoses [Fig. 5]. The number of participants with PKU who reported having at least one skin condition was 99 (46%). [Figure 6] shows the number of skin conditions reported (Range = 40; Median = 3).

### Resources and professional support

The resources developed by NBS Connect’s operational team were recognized by parents as useful tools, particularly the *Kitchen Connection*. This feedback was received during the consumer session of the southeast regional meeting and a data collection tool will be implemented for ongoing evaluation. Based on participant feedback to increase consumer interaction, *Kitchen Connection* now includes a *Recipe of the Month* feature. Registry members can submit their own recipes to be reviewed and analyzed by a Registered Dietitian Nutritionist (RDN), and included on the website with credit to the contributor.

Patients have expressed that the *Ask an Expert* tool is particularly useful when they have a question between clinic visits. Response time and patient satisfaction with question responses will be assessed in the future.

A discussion forum, created to increase consumer interactions and participant connection with other registered users who may face the similar daily challenges, is reported to be useful by patients and families.

The interactive health tracking tool allows participants to assess their responses to profile questions compared to the responses of others. Participants can access the user friendly explore tab to view de-identified aggregate data as charts and graphs. This tool gives families a visual representation of data on care and treatment strategies used by NBS Connect participants and provides a comparative self-assessment with other members of the community.

## Discussion

NBS Connect is the first patient registry for individuals and families affected by IMD, as well as caregivers and metabolic clinicians. The self-reported registry provides privacy and eliminates the need to divulge personal information to a study coordinator. Self-reporting also provides a much larger sample size for research endeavors, allowing participation from individuals with IMD around the world.

NBS Connect is relevant to patients, parents and family members of those with IMD. The NBS Connect website is user-friendly and welcoming to patients and parents, developed with direct input from consumers. The website fosters participation and interaction between patients/families and clinicians, and connects individuals who may otherwise feel very isolated in living with a rare disorder.

Within two years of its launch, NBS Connect has shown great success, registering close to 450 individuals with 24 different metabolic disorders. Disorder-specific profiles for Homocystinuria, Maternal PKU and FAOD are under development, and additional disease profiles will be created over time. Unfortunately, individuals with rare disorders may lack financial or organizational support to start independent support networks. NBS Connect fills this gap by serving as a common platform for patients, families and stakeholders. NBS Connect social media platforms such as Facebook and Twitter are also utilized to keep participants engaged.

### Application of data

NBS Connect collects information on current diagnosis, management, and treatment practices. De-identified patient data allows participants, clinicians and researchers to monitor patients’ health over time with annual profile updates, and provides an opportunity for research on rare disorders [11].

Data collected on age of diagnosis show that NBS identifies the majority of affected individuals at birth, but there are still children who are undiagnosed until they show symptoms. Examining demographic information, such as state of residence in late-diagnosed individuals, shows where improvements in screening and diagnosis are needed.

NBS Connect also collects data on health indicators including bone health and skin conditions. Individuals with PKU are reported to have bone density issues, hypothesized to result from long term use of protein restricted diets and possible calcium deficiencies [12–16]. Data collected through NBS Connect show a discrepancy in the number of individuals who had a DXA scan versus those who did not. The Nutrition Management Guidelines for individuals with PKU provides frequency recommendations for monitoring and assessment of bone metabolism abnormalities and bone density testing by age group. NBS Connect data can be used to evaluate these recommendations in actual patients, revealing the gap between evidence-based recommendations and actual clinical care. [17]

Preliminary data collected through NBS Connect also show an over-representation of individuals with skin conditions (69%) [18, 19]. A study reported that high phenylalanine levels can inhibit biotinidase activity, causing some skin conditions [20], but the association between the two has not been examined. Observational data from NBS Connect, may support this hypothesis and the need for additional research is indicated to confirm these observations. Also, since there is no comparison group, it may be that these self-reported skin problems are quite common in young people generally.

Given the absence of information on long-term outcomes in individuals with inherited metabolic disorders, NBS Connect’s ability to capture participants’ health status prospectively is a substantial gain to the scientific community. Assessment of long-term outcomes has broad implications for future research and treatment strategies. By tracking the natural history of registered participants, a longitudinal understanding of disease progression will now be possible.

#### Benefits and limitations

The NBS Connect patient registry has many advantages as well as some limitations. This registry is intended to have a large population base which is not restricted by state, region or country. The global reach allows large numbers of patients to register and gain access to tools, resources, professional support, and receive updates on clinical trials and research for which they may be eligible. Offering registration at clinic visits will broaden participation, including those that may not have access to the necessary technology for online registration and data input.

Experts such as RDNs and public health professionals are closely involved in the day-to-day administration of the registry, ensuring data are accurate. Finally, the standardized format of basic and disorder-specific profiles for all conditions allows comparisons across disorders [21].

Along with the immediate benefits for patients and families, there are tremendous long term benefits. By collecting data on diagnosis, treatment, symptoms, outcomes, barriers to care, and quality of life, we hope to eliminate gaps in service and improve access to care while establishing best standards of practice for clinical management of all IMD [22, 23].

NBS Connect is limited as a self-reported system. Missing or incomplete data are a possibility when relying on patient entry; however, data are curated and monitored by the operational team to verify submitted information is accurate.

Despite efforts in developing and distributing outreach materials, delivering oral and poster presentations at consumer and professional conferences, meetings and symposiums, and creating a social media presence to bolster exposure, the number of participants continues to be less than expected. Since enrollment is low, resources provided are under-utilized. Also, the majority of registered participants are patients recruited through the Emory Metabolic Clinic in Atlanta, Georgia, the location of the NBS Connect operational team; therefore there is an over-representation of participants from Georgia, leading to limited diversity.

Although the NBS metabolic community consists of many rare disorders, the total number of patients living with disorders is fortunately low. With the introduction of additional disorder-specific profiles, the number of participants enrolled is projected to increase. NBS Connect has a goal to reach all affected individuals. With that in mind, we continue to explore additional strategies to increase awareness including visiting other clinics in the southeast region, nationally and potentially globally.

## Conclusion

Fifty years have passed since the launch of NBS, yet there are insufficient resources for many rare heritable conditions. NBS Connect is the first self-reported patient registry for NBS disorders, with an initial focus on IMD [24].

Participation in NBS Connect provides motivation for patients to provide data, access to critical resources, and opportunities for connections and support with clinicians and peers. Patients who may have previously felt isolated can engage in an entire network of individuals sharing similar social, physical, and emotional concerns. NBS Connect fosters a powerful sense of community and motivation, otherwise lacking in the community of individuals with disorders included on the NBS panel.

Since its launch in 2012, NBS Connect has offered novel benefits to both the patient and scientific communities. Moreover, data collected by the registry have been used to develop hypotheses to guide scientific research, an excellent example of translational medicine. Most importantly, the database will continue to be curated by professionals such as genetic counselors and dietitians to inform clinical practice and drive medical research.

In conclusion, patient registries like NBS Connect can inform research needs and hypothesis-driven research. Data collected through NBS Connect is integral in understanding long-term outcomes of very rare disorders. Ultimately, registries like NBS Connect will yield a better understanding of long-term outcomes of rare disorders, leading to better patient care and improved quality of life.

## Abbreviations

ACMG: American College of Medical Genetics and Genomics
CDC: Centers for disease control and prevention
DXA: Dual energy x-ray absorptiometry
FAOD: Fatty acid oxidation disorder
GMDI: Genetic metabolic dietitians international
HIPAA: Health insurance portability and accountability act
HRSA: Health resources and services administration
IMD: Inherited metabolic disorders
MSUD: Maple syrup urine disease
NBS Connect: Newborn screening connect patient registry
NBS: Newborn screening
NBSTRN: Newborn screening translational research network
NIH: National institutes of health
NPKUA: National PKU alliance
NSGC: National society of genetic counselors
OAA: Organic acidemia association
PHI: Protected health information
PI: Principle investigator
PKU: Phenylketonuria
RDN: Registered dietitian nutritionist
RUSP: Recommended uniform screening panel
SERC: Southeast regional newborn screening & genetics collaborative
SSL: Secure sockets layer
TYR: Tyrosinemia
URL: Uniform resource locator
VPN: Virtual private network
